# Influence of ozonized water on pain, oedema, and trismus during impacted third molar surgery: a randomized, triple blind clinical trial

**DOI:** 10.1186/s12903-020-1029-5

**Published:** 2020-02-05

**Authors:** José Cristiano Ramos Glória, Dhelfeson Willya Douglas-de-Oliveira, Larissa Doalla Almeida e Silva, Saulo Gabriel Moreira Falci, Cássio Roberto Rocha dos Santos

**Affiliations:** Departament of Dentistry, Faculty of Biologic Sciences and Health, Federal University of Jequitinhonha and Mucuri Valleys, Rua da Glória, 187, Centro, Diamantina, Minas Gerais Brazil

**Keywords:** Ozone, Third molar, Oral surgery, Pain, Swelling, Trismus

## Abstract

**Background:**

This study aimed to evaluate the efficacy of ozonized water on pain, oedema and trismus after impacted third molar mandibular surgeries when compared to double distilled water. A randomized triple blind trial was conducted.

**Methods:**

Patients with third molars class II-B of Pell-Gregory were included, and surgical extraction was performed. Irrigation was done with ozonized (group 1) or double distilled water (group 2). The type of irrigation and the side to be operated were randomized. Neither the patients nor the operator or evaluator were aware of the irrigation solution. Pain, oedema and trismus were evaluated at baseline, 24-h, 48-h, 72-h and 7-days after treatment. The data were evaluated by Friedman, Wilcoxon, Mann-Whitney tests, and size effect.

**Results:**

It was included 8 men and 12 women, with a mean age of 20.9y.o. The initial pain mean was 7.94 (±12.81) (group 1) and 5.50 (±9.12) (group 2) (*p* > 0,05). There was a statistically significant reduction of pain, oedema and trismus in intragroup analysis (*p* < 0.05). There was no statistically significant difference (*p* > 0.05) when comparing the oedema and trismus between groups. The size effect ranged from small (0.23) to large (1.29).

**Conclusions:**

It was concluded that ozonized water was compatible as irrigation method, not inferior to double distilled water, and had satisfactory effects on management of pain, oedema and trismus after surgical removal of the third molar.

**Trial registration:**

This clinical trial was registered in ClinicalTrials.gov NCT03501225 on April 18, 2018.

## Introduction

Ozone therapy is a modern, non-medicated alternative to control post-operative complications [1]. The antibacterial properties of ozone, as well as its efficacy in the treatment of infection, and hemodynamic and anti-inflammatory properties have been demonstrated [2]. The advantages of this treatment are: simplicity of execution, good tolerance of patients, absence of side effects or adverse reactions and high medical-social efficiency [1].

The application of ozone in Dentistry is based on its antimicrobial properties against gram positive and negative bacteria, viruses and fungi [3]; based on cellular biological characteristics in terms of biocompatibility for oral application [4]. Ozonized water applied daily may accelerate the epithelial healing of the oral cavity, especially on the first two post-operative days [5]. Ozonized water (0.5–4 μg / mL) was highly effective in killing gram positive and negative microorganisms present in the dental biofilm, in addition to inhibiting the accumulation of experimental plaques in vitro [6]. Ozonized water also produces less cytotoxicity than ozone gas, chlorhexidine 0.2 and 2%; sodium hypochlorite 2.25 and 5.25%; hydrogen peroxide 3% [4].

Extraction of third molars is one of the most accomplished procedures in oral surgery [7]. Indications for this surgery include caries, pericoronitis, periodontal problems and cysts formation [7–9]. Depending on the location, depth, tooth angulation and bone density, the complexity of surgical extraction may vary, and is generally associated with post-operative pain, oedema, and trismus [10].

Studies have emphasized the need to improve the control of pain, oedema and trismus on these patients in an attempt to improve their quality of life after surgical procedures [11–14]. Studies have verified the pre and post-operative effects of different drugs and /or clinical management to control these parameters [7, 10, 13].

Recently, some strategies have been developed for minimizing postoperative discomfort after third molar surgery, including the use of pharmacological therapy and alternative medicine [15, 16], and complementary protocols have been suggested for the postsurgical therapy of third molar surgery [16]. However, there is a gap about the effect of the trans-operative irrigation solution on the post-operative discomfort management. In light of these findings, the objective of this study was to evaluate the effect of ozonized double-distilled water compared to double-distilled water, as irrigation methods, on the postoperative pain, oedema and trismus after third molar surgery. The null hypothesis was that there would be no difference between the two irrigation solutions analysed.

## Materials and methods

It is a randomized, triple blind trial using the split-mouth model. Patients were referred for treatment at the Surgery and Periodontics Clinic of the Federal University of the Jequitinhonha and Mucuri Valleys (UFVJM), Brazil. This study was approved by the Research Ethics Committee of UFVJM (# 2174074) and was carried out according to the Declaration of Helsinki of 1975, revised in 2013. All subjects signed a written informed consent form before the beginning of this study. This clinical trial was enrolled in ClinicalTrials.gov (NCT03501225), and developed according to CONSORT.

### Training and calculation

Training and clinical calibration were performed for face and mouth opening measurements. A single investigator (LDAS) was calibrated at the Periodontics and Oral Surgery Clinic of the UFVJM, using 10 patients (not included in the research) through the test-retest with a 15-days interval. The intra-class correlation coefficient was 0.886 and 0.814 respectively.

### Sample calculation

The sample size was calculated for comparison of mean values, which considered standard deviation of pain 3.32 mm [5] and the difference to be detected between groups stipulated in 3 mm on the visual analog scale. Level of significance of 95% and power of test of 80% were applied, determining 19 patients. Two patients were added to cover any dropouts. Patients were recruited between November 2017 and February 2018.

### Eligibility criteria

The inclusion criteria were as follows: (1) age between 18 and 30 years; (2) good general health; (3) the presence of bilateral mandibular third molar with class II position, type B impaction according to Pell-Gregory [6]. The exclusion criteria were: (1) any systemic condition which might contraindicate the ozone therapy; (2) status of pregnancy or lactation; (3) acute hemorrhage during the last 30 days; (4) pericoronitis or signs of infection/inflammation; and (5) smoking habit [17].

A pre-operative X-ray was requested. Orthopantomography was used to determine tooth position. Surgeries were scheduled in two separate clinical sessions, at least 4 weeks apart.

### Allocation of volunteers, randomization and sequence generation

The patients selected were numbered from 1 to 21. Through a drawing, group 1 was assigned ozonized water (test group) and group 2 double distilled water (control group). The volunteers were randomly assigned to one of the two groups using an accurate dice; if an odd number was drawn, the subject was allocated to group 1; and even number to group 2. The side to be operated on was also randomized by an accurate dice; odd number, indicated right-sided surgery; even number, left side. In this sequence, the data was released twice per patient.

The results of the sweepstakes were placed inside two opaque envelopes, sealed and duly identified with the patient’s code.

### Blinding and allocation concealment

Information on the type of intervention was unknown by the patient, surgeon, clinical investigator (patient follow-up and outcome measures) and statistician. The allocation of the interventions was kept secret by a collaborator (JNS) external to the study that was unaware of the research protocol. The opaque and sealed envelopes were stored, and opened only 5 min before the beginning of the surgery at the preparation area of the irrigating solution (ozonized or double-distilled water).

### Ozonized double-distilled water

Ozonized double-distilled water was prepared 5 min before the surgeries by an external collaborator of this research (JNS); using the model MedPlus ozone generator (Philozon®, Santa Catarina, Brazil) coupled to the glass column with a catalytic converter and microbubble diffuser in stainless steel tube. Double-distilled water absorbs 20 to 25% of all the concentration that is offered to it [18]. The ozone generator was regulated at 40 μg / mL for 5 min of bubbling in 250 ml of double-distilled water (Sanobiol, Pouso Alegre, Brazil). The final concentration was 8.0 μg / mL.

### Double-distilled water

Double-distilled water (clear, hypotonic, sterile and pyrogenic solution), 250 ml bottle, was purchased from the manufacturer Sanobiol.

### Outcome measures

Measurements were collected and stored in an envelope, avoiding the access of the evaluator to the previous measures.

To evaluate the intensity of post-operative pain, a horizontal visual analogue scale of 10 cm was used, without demarcations, representing the left extremity without any pain, and the right extremity the maximum pain. The patients were instructed to mark with a vertical line the point of the scale that best defined the degree of pain at 24, 48 and 72 h after the surgical procedures. These markings were measured with a digital caliper (Mitutoyo®) with two decimal places.

Oedema was determined by measuring with measuring tape according to the method described by angulatura [19]. Three measures were taken: 1. corner of the eye to the angle of the mandible, 2. tragus to the corner of the mouth, 3. tragus to the pogonium. The measurements were obtained in the pre and post-operative periods of 24, 48 and 72 h and 7 days. The sum of the pre-operative measurements was considered the standard of normality for each side. After verification of the measurements from the post-operative period, the difference between the measurements before and after the surgical procedure was observed, determining the level of oedema.

Trismus was evaluated by maximum buccal opening. The maximum inter-incisional vertical distance was measured using a digital caliper (Mitutoyo®) with two decimal places and transcribed in millimeters. The measurement considered the distance between the incisal surfaces of the upper and lower right central incisors, after maximum opening without aid, pre-operatively and post-operatively 24, 48, 72 h and 7 days. The amount of reduction of the buccal opening was measured and compared to the baseline. Thus, the relative mean for each patient between the final and initial measurements (Delta) was calculated.

Surgeries were timed from the moment of the incision until the final suture.

### Surgical intervention

The surgery was performed in an outpatient setting by a single professional (JCRG) with experience in oral surgery under strict control within the biosafety norms. In order to reduce the differences in intraoperative trauma level, the same surgical procedure was adopted for both sides.

Iodized polyvinylpyrrolidone-iodine alcohol solution at 10% was used in extra oral antisepsis. Regional inferior and lingual alveolar nerves block was performed, with complementation of the buccal nerve anesthesia. A maximum volume of up to 5.4 mL of anesthetic solution (2% lidocaine and 1: 100,000 epinephrine) was used for each surgery.

The surgeries performed in all cases can be described as follows: after incision of Avellanal (1946) with a scalpel blade number 15 and removal of the soft tissues to expose the surgical area, osteotomy at low rotation (30,000 rpm) was performed with a drill 8 and (350,000 rpm) with carbide 702C KGS drills (straight piece and pen / Dabi Atlante®), under constant irrigation using a 10 cc (ruthe) glass hypodermic syringe coupled to a 10 mm (BD) steel needle with doubly distilled water or ozonized water according to randomization. Then, the third molars were extracted with the help of straight Seldin lever, careful curettage, bone regularization and cleaning of the surgical area by means of abundant irrigation followed by aspiration. Suture performed with silk thread (4.0), through isolated stitches, and removed after 7 days.

After each surgery the patients received postoperative instructions and were prescribed sodium dipyrone (500 mg), 1 tablet every 6 h in case of painful symptomatology; and nimesulide (100 mg), 1 tablet every 12 h for 3 days.

### Statistical analysis

The results were typed and analyzed using the Statistical Package for Social Science software, version 25. This process was carried out by two people (one typed and one checked). The envelope containing the data of each patient received a corresponding number in the database. Statistical analysis was initially performed with groups coded as group 1 and group 2. The decoding envelope of this information was accessed upon completion of the clinical trial and statistical analysis. Descriptive statistics were analyzed to obtain mean and standard deviation. Data normality was verified by the Shapiro-Wilk test. The association between variables was verified by Mann-Whitnney and Wilcoxon tests, when pertinent. The significance level of 95% (*p* < 0.050) was considered.

To check the magnitude of the differences obtained between the baseline and the last evaluation, the magnitude of effect was analyzed for each group according to the variable. It was used the Cohen’s d to calculate the size effect of these dependent variables. The results were categorized as having a small (0.20 < *d*), medium (0.21 < *d* < 0.50), or large (*d >* 0.51) effect [20].

## Results

**Fig. 1:**
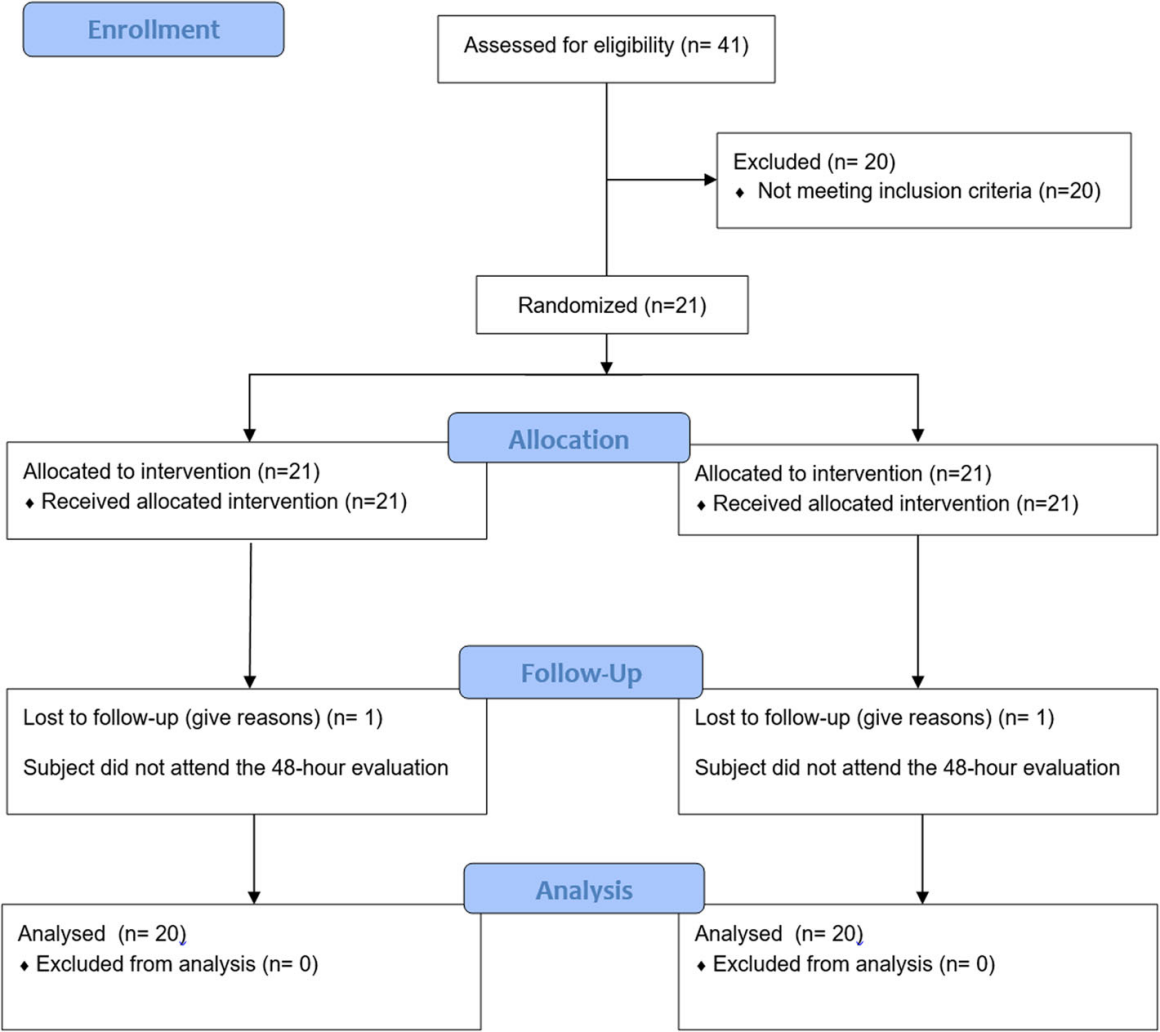
Flowchart of the included subjects

This study included 8 men, and 12 women, with a mean age of 20.9 years. Of the 41 adult volunteers evaluated, 20 did not fit the inclusion criteria, and 1 did not attend the 48-h post-operative period and was excluded from the study. At the end, 20 participants completed the entire research protocol (Fig. 1). There were no intercurrences. Twenty impacted teeth were included in each group. The mean time of surgery was 15.65 (±6,94) minutes for group 1, and 15.90 (±5,56) minutes for group 2. There was no statistical difference when the mean time between groups was compared (*p* = 0.794).

In the 24 h evaluation, the mean pain level of the group 1 and 2 was 7.94 (±12.81) and 5.50 (±9.12) respectively. At 72 h, the pain reduced for 5.67 (±12.83) in group 1, and 8.58 (±10.45) in group 2. There was no statistically significant difference between the groups in the different evaluation times (*p* > 0.05). In the intragroup analysis, no statistically significant difference was observed between the post-operative times (*p* < 0.05) (Table 1).

**Table 1:** Evolution of mean of the pain scores obtained by VAS

Pain	Time	Mean (sd)	p^a^	p^b^
Group 1	24 h	7.94 (12.81)	24 h × 48 h 0.278	24 h 0.653 48 h 0.948 72 h 0.267
	48 h	4.73 (5.62)	24 h × 72 h 0.246	
	72 h	5.67 (12.83)	48 h × 72 h 0.814	
Group 2	24 h	5.50 (9.12)	24 h × 48 h 0.948	
	48 h	5.50 (9.43)	24 h × 72 h 0.629	
	72 h	8.58 (10.45)	48 h × 72 h 0.841	

^a^Intragroup analysis

^b^intergroup analysis

The oedema reached its peak at the 24-h post-operative assessment (38.35 ± 1.38 in the group 1; 38.70 ± 2.07 in the group 2) (p < 0.05), and the facial contour began to resume normal at the 7-days evaluation (37.95 ± 1.54 in the group 1; 38.06 ± 1.99 in the group 2). No statistically significant differences were observed between groups at all post-operative times (*p* > 0.05) (Table 2).

**Table 2:** Evolution of mean of the oedema scores

OEDEMA	Time	Mean (sd)	p^a^	p^b^
Group 1	Baseline 24 h 48 h 72 h 7 days	37.17 (3.13) 38.35 (1.38) 38.33 (1.47) 38.25 (1.66) 37.95 (1.54)	Baseline × 24 h 0.003 Baseline × 48 h 0.045 Baseline × 72 h 0.028 Baseline × 7 days 0.255 24 h × 48 h 0.999 24 h × 72 h 0.549 24 h × 7 days 0.138 48 h × 72 h 0.588 48 h × 7 days 0.164 72 h × 7 days 0.147	Baseline 0.142 24 h 0.325 48 h 0.162 72 h 0.474 7 days 0.589
Group 2	Baseline 24 h 48 h 72 h 7 days	38.07 (2.04) 38.70 (2.07) 38.01 (2.91) 38.01 (2.14) 38.06 (1.99)	Baseline × 24 h 0.085 Baseline × 48 h 0.078 Baseline × 72 h 0.704 Baseline × 7 days 0.550 24 h × 48 h 0.925 24 h × 72 h 0.068 24 h × 7 days 0.172 48 h × 72 h 0.004 48 h × 7 days 0.072 72 h × 7 days 0.875	

^a^Intragroup analysis

^b^intergroup analysis

The initial mean of mouth opening was 47.46 (±6.92) in group 1 and 48.47 (±7.22) in group 2. At 7 days, this opening was 49.49 (±11.18) and 41.46 (±7.90) in group 1 and 2 respectively. It was observed a significant reduction of the buccal opening in all post-operative periods compared to the baseline, regardless of the type of irrigation used (*p* < 0.05). However, no statistically significant difference was observed when comparing the oral opening between groups (*p* > 0.05) (Table 3).

**Table 3:** Evolution of mean of the trismus scores

TRISMUS	Time	Mean (sd)	p^a^	p^b^
Group 1	Baseline 24 h 48 h 72 h 7 days	47.46 (6.92) 35.71 (12.08) 36.74 (12.90) 38.93 (10.37) 39.49 (11.18)	Baseline × 24 h < 0.001 Baseline × 48 h < 0.001 Baseline × 72 h < 0.001 Baseline × 7 days 0.003 24 h × 48 h 0.136 24 h × 72 h 0.030 24 h × 7 days 0.005 48 h × 72 h 0.094 48 h × 7 days 0.084 72 h × 7 days 0.421	Baseline 0.126 24 h 0.601 48 h 0.469 72 h 0.421 7 days 0.433
Group 2	Baseline 24 h 48 h 72 h 7 days	48.47 (7.22) 35.89 (9.17) 37.81 (9.50) 38.28 (10.86) 41.46 (7.90)	Baseline × 24 h < 0.001 Baseline × 48 h < 0.001 Baseline × 72 h < 0.001 Baseline × 7 days 0.001 24 h × 48 h 0.064 24 h × 72 h 0.171 24 h × 7 days 0.005 48 h × 72 h 0.687 48 h × 7 days 0.008 72 h × 7 days 0.053	

^a^Intragroup analysis

^b^intergroup analysis

Delta trismus in 24 h evaluation was − 11.75 (±10.30) in group 1 and − 12.57 (±7.35) in group 2. In both groups, there was a statistically significant difference between the evaluation of 24 h and 7 days (*p* = 0.005). There was no statistically significant difference in delta trismus between groups (p > 0.05) (Table 4).

**Table 4:** Difference of evaluation time minus baseline trismus measurement

Δ TRISMUS	Time	Mean (sd)	p^a^	p^b^
Group 1	24 h 48 h 72 h 7 days	− 11.75 (10.30) − 10.70 (9.59) − 8.53 (7.18) − 7.48 (9.48)	24 h × 48 h 0.136 24 h × 72 h 0.030 24 h × 7 days 0.005 48 h × 72 h 0.094 48 h × 7 days 0.084 72 h × 7 days 0.421	24 h 0.355 48 h 0.841 72 h 0.398 7 days 0.573
Group 2	24 h 48 h 72 h 7 days	− 12.57 (7.35) -10.65 (8.23) − 10.19 (9.17) − 7.01 (7.87)	24 h × 48 h 0.064 24 h × 72 h 0.171 24 h × 7 days 0.005 48 h × 72 h 0.687 48 h × 7 days 0.008 72 h × 7 days 0.053	

^a^Intragroup analysis

^b^intergroup analysis

In both groups, the effect size was large for trismus (1.19 in group 1; 1.29 in group 2) and medium for pain (0.23 in group 1; 0.43 in group 2) (Table 5).

**Table 5:** Effect size of the pre and post treatment

	Cohen’s d	
	Group 1	Group 2
Pain	0.23	0.43
Edema	0.43	0.01
Trismus	1.19	1.29
Δ TRISMUS	0.60	1.02

## Discussion

=No previous studies evaluating the effect of ozonized double distilled water (trans operative) on pain, oedema, and trismus after third molar mandibular surgery were found. Considering ozone therapy a non-drug therapy and with no side effects, it is justified to use it in invasive procedures, such as third molar removal surgery.

In order to avoid inter-patient bias in the collection of the pain, oedema and trismus levels, the split-mouth model was used. Thus, each patient was his/her own control [18] which increases the statistical efficiency. This way, control and study groups were submitted to the same conditions of feeding, stress, healing, habits (routines and addictions).

Previous studies used ozone therapy through gel applied in the extra oral region immediately after removal of lower third molars, 1, 3, 5 and 7 post-operative days did not show the efficacy of ozone in reducing edema and trismus [5]. Ozonized gel applied to the surgical wound for 2 min, twice daily for 3 days, decreased operative pain, oedema and trismus [17]. Ozonized gel, contains stable ozonide which, when in contact with the wound (body temperature), decomposes to reactive active ozone in a prolonged way [21]. We believe that the application of ozone coincided with the peak of inflammation, which may have significantly reduced post-operative oedema in the study group [21]. The present study applied ozonized double distilled water only in the trans-operative period, with no further post-operative complementation, which may have influenced this result.

The literature shows that the time elapsed in surgery correlates significantly with pain, trismus and total analgesic consumption [22]. The present study presented homogeneity of the data between the groups, due to the absence of statistically significant differences in sex, age, dental position, bone removal and / or dental sectioning, duration of surgery, mean time on the operated side and post-operative bleeding, which indicates comparability between groups and validity of the results. In addition, a single surgeon performed all surgical procedures to avoid interpersonal differences, which could influence the results.

Previous in vitro study used ozonized water with the following concentrations: 2 μg / mL, 5 μg / mL and 8 μg / mL and concluded that 8 μg / mL were more efficient in eliminating *Pseudomonas aeruginosa*, *Staphylococcus aureus*, and *Enterococcus faecalis* [18]. The antibacterial capacity of the ozonized double-distilled water may have contributed to avoiding post-operative infections in the present study.

The present results showed that 8.0 μg / mL of ozonized double distilled-water had no significant impact on pain reduction, oedema and trismus in the post-operative period, when compared to the control group, however, on the intragroup analysis; it was efficient and not less than double distilled water. A suggested explanation for this result would be the reduced contact time of the ozonized double distilled water with the surgical area, bone and soft tissues. Studies have shown that the effectiveness of ozonized water depended on several factors such as: contact time, PH and temperature [23]. Ozone destroys microorganisms by the progressive oxidation of vital cellular components. The accumulation of the oxidation effect due to a longer contact time of the ozonized water contributes to a greater reduction of microorganisms [24].

The interval between surgeries in similar studies, 2 weeks [10] and 3 weeks [17] was extended in the present study to 4 weeks in order to guarantee complete healing of the wound and to eliminate overlap of symptoms as well as distortion of the results. This favored the study because, with a longer interval between surgeries, the conditions of the first surgery did not influence the second.

As discussed above, the present study was well conducted, presenting internal and external validity.

Surgical removal of the third molar is followed by the release of various inflammatory mediators which results in an increase in vascular changes, leading to peripheral oedema and local tissue alterations, like pain and trismus [25]. Non-steroidal anti-inflammatory drugs and corticosteroids are the most commonly used drugs in post-surgical period, since they play important role in the management of postoperative complications. The present study prescribed a commonly used anti-inflammatory drug in both groups. Isola et al. (2019) [15] investigated the efficacy of celecoxib and ibuprofen in reducing postoperative sequelae following the surgical removal of impacted mandibular third molars, and they concluded that celecoxib shows favourable effects in the management of perioperative pain.

Pain is caused by inflammation from tissue injury and the release of pain mediators [26], however, causal factors are not exclusively dependent on the procedure, but a part is also attributed to the patient’s physiological capacity as well as his/her level of anxiety [27]. Pain is considered a common consequence in the post-operative period of dental extraction, followed by a decrease over the days [27]. The present result confirms this transitory character, probably due to biosafety, professional experience, post-operative guidelines and patient health.

Oedema usually occurs in response to tissue trauma on the third molar area. Its evolution is gradual, with a peak of oedema within 48 h after surgery [28]. However, it has been reported that oedema may increase on the third day, and remain for up to 7 days [29]. The present study corroborates data from the literature that demonstrates an initial edema that resolves within 1 week. The ozonized double-distilled water was as equally effective as double-distilled water for oedema control.

Trismus usually reaches its peak on the second post-operative day and resolves at the end of the first week [30]. The present findings indicate that there was a significant reduction on mouth opening in 24 h and that it was maintained 7 days after surgical procedure. This trismus in both groups can be attributed to the presence of oedema, injury to muscle fibers, multiple penetrations of the needle, elevation and manipulation of flaps, and presence of anesthetic or clot in the muscle fibers.

During the third molar removal surgery, trans-operative irrigation avoids bone lesions, tissue overheating, and improves operator vision [31]. Saline solution is the most commonly used as an irrigating solution for the surgical removal of third molars; however, there are alternatives, for example, sterile water [31]. In the present study, double-distilled water (sterile water) was used as a control because of its easy availability, low cost, non-irritant, non-toxic, non-hemolytic and antiseptic.

The saline solution is isotonic and has similar physiological properties to the natural tissue fluid, however, it is discussed that ozonized saline can produce hydrogen peroxide (H_2_O_2_) and chloride ions [2]. Therefore, ozonized double-distilled water was chosen as a test irrigation method because of its safety, non-by-product formation and easy manipulation. As explained, double-distilled water was used as the control so that the ozone effect (ozonized double-distilled water) could be evidenced, avoiding the bias of the irrigating solution.

It was expected that the ozonized water could present better result as irrigation solution, since studies have shown that ozone has good effect/interaction with tissues when exposed in the long run. However, it was such effective as the control group. This may be explained by the fact that the present ozonized water was immediately aspirated after irrigation, its contact with the tissues was reduced, consequently inhibiting its effectiveness. This new irrigation solution during surgery showed to be not inferior to double-distilled water.

Although there was no significant difference between ozone and double-distilled water in the evaluation of pain, oedema, and trismus, both agents were clinically efficient. Clinical decision-making based on the value of p has been questioned, and clinical significance seems to be more focused on the reality of practical and immediate use [32]. Cohen’s d analysis showed clinical importance ranging from medium to large. This implies that in daily practice, ozonized double-distilled water is able to provide good results on the control of pain, oedema, and trismus. However, double-distilled water presented the same behavior of clinical importance.

Significant information could not be found in literature on the use of ozonized double-distilled water during tooth removal. The clinical trial was well delineated, under extreme methodological rigor, however, a limitation of the present study was to find no publications on the use of ozonized water in oral surgical procedures; being this, a pioneering study, the results can be preliminary.

The present study showed a novel and viable irrigation solution for third molar surgery. Similarly, the study of Isola et al. (2019) [16] reported interesting results regarding the management of pain, facial swelling and trismus after third molar removal by using a phytotherapeutic drug contained a mixture of herbal extracts such as baicalin (190 mg), bromelain (50 mg), and escin (30 mg). This emphasize the importance of researches in order to evaluate the potential impact of new treatment strategies and alternative drugs linked with postsurgical healing following third molar surgery.

In view of the present, more studies are needed to confirm the effectiveness of ozonized double-distilled water in the reduction of pain, oedema, and trismus in surgery of impacted lower third molars. Further studies with the use of ozone in gel and / or gas are suggested, as well as the time of contact of the ozone with the tissues should be made longer. It is also suggested to investigate the effect of ozone on short- and long-term pain, for example by measuring the number of analgesics ingested.

## Conclusion

In conclusion, the ozonized double-distilled water, when used as a trans-surgical irrigation solution, demonstrated to be viable and safe, presenting satisfactory effects on management of pain, oedema and trismus after surgical removal of the third molar.

## Data Availability

Data will be available upon request by email.

## Abbreviations

mL: Milliliters
UFVJM: Federal University of the Jequitinhonha and Mucuri Valleys
μg: Micrograms
